# Chirped pulse amplification in an extreme-ultraviolet free-electron laser

**DOI:** 10.1038/ncomms13688

**Published:** 2016-12-01

**Authors:** David Gauthier, Enrico Allaria, Marcello Coreno, Ivan Cudin, Hugo Dacasa, Miltcho Boyanov Danailov, Alexander Demidovich, Simone Di Mitri, Bruno Diviacco, Eugenio Ferrari, Paola Finetti, Fabio Frassetto, David Garzella, Swen Künzel, Vincent Leroux, Benoît Mahieu, Nicola Mahne, Michael Meyer, Tommaso Mazza, Paolo Miotti, Giuseppe Penco, Lorenzo Raimondi, Primož Rebernik Ribič, Robert Richter, Eléonore Roussel, Sebastian Schulz, Luca Sturari, Cristian Svetina, Mauro Trovò, Paul Andreas Walker, Marco Zangrando, Carlo Callegari, Marta Fajardo, Luca Poletto, Philippe Zeitoun, Luca Giannessi, Giovanni De Ninno

**Affiliations:** 1 Elettra-Sincrotrone Trieste, Strada Statale 14-km 163,5, 34149 Trieste, Italy; 2 CNR-ISM, Trieste, Basovizza Area Science Park, 34149 Trieste, Italy; 3 Laboratoire d'Optique Appliquée, ENSTA ParisTech - CNRS UMR, 7639 Palaiseau, France; 4 CNR - Institute of Photonics and Nanotechnologies, via Trasea 7, 35131 Padova, Italy; 5 Commissariat l'Energie Atomique et aux Energies Alternatives - DRF/IRAMIS/LIDYL, Centre d'Etudes de Saclay, 91191 Gif sur Yvette, France; 6 Instituto de Plasmas e Fusão Nuclear, Instituto Superior Técnico, 1049-001 Lisboa, Portugal; 7 Center for Free-Electron Laser Science and Department of Physics, University of Hamburg, Luruper Chaussee 149, 22761 Hamburg, Germany; 8 ELI-Beamlines, 252 41 Dolní Břežany, Czech Republic; 9 European XFEL GmbH, Albert-Einstein-Ring 19, 22761 Hamburg, Germany; 10 Max Planck Institute for the Structure and Dynamics of Matter, Luruper Chaussee 149, 22761 Hamburg, Germany; 11 IOM-CNR, 34149 Trieste, Italy; 12 Enea, via Enrico Fermi 45, 00044 Frascati, Roma, Italy; 13 Laboratory of Quantum Optics, University of Nova Gorica, 5001 Nova Gorica, Slovenia

## Abstract

Chirped pulse amplification in optical lasers is a revolutionary technique, which allows the generation of extremely powerful femtosecond pulses in the infrared and visible spectral ranges. Such pulses are nowadays an indispensable tool for a myriad of applications, both in fundamental and applied research. In recent years, a strong need emerged for light sources producing ultra-short and intense laser-like X-ray pulses, to be used for experiments in a variety of disciplines, ranging from physics and chemistry to biology and material sciences. This demand was satisfied by the advent of short-wavelength free-electron lasers. However, for any given free-electron laser setup, a limit presently exists in the generation of ultra-short pulses carrying substantial energy. Here we present the experimental implementation of chirped pulse amplification on a seeded free-electron laser in the extreme-ultraviolet, paving the way to the generation of fully coherent sub-femtosecond gigawatt pulses in the water window (2.3–4.4 nm).

## Abstract (web summary)

Short laser pulses of femtosecond time scales are in high demand in order to explore the fast electron dynamics in light-matter interactions. Here, the authors demonstrated the compression of free electron laser pulses in the extreme ultraviolet range by using a chirped pulse amplification technique.

## Graphical abstract

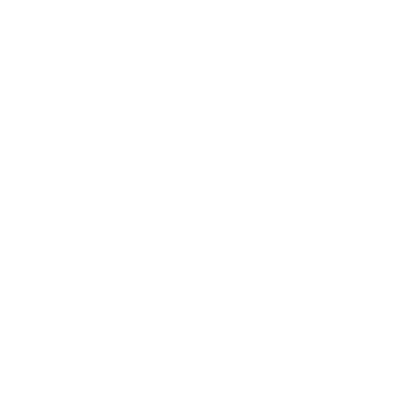

Several methods have been proposed to generate ultra-short pulses in short-wavelength free-electron lasers (FELs)^1^,^2^,^3^,^4^,^5^. In these methods^6^,^7^,^8^,^9^,^10^, the output pulse energy is limited by the reduced number of electrons participating in the amplification process. More recently, few theoretical schemes have been proposed to increase the peak power up to the terawatt level^11^,^12^. However, in all cases, the pulse shortening is constrained by the FEL gain bandwidth. This represents an obstacle to the investigation of ultra-fast reaction mechanisms, occurring both in dilute and solid matter, with a nanometric resolution. Moreover, none of the proposed methods allows to control the spectro-temporal properties of the generated light, which is a fundamental requisite for the production of fully coherent optical pulses^13^.

An alternative process for generating ultra-short pulses with high peak power relies on laser harmonic generation in gas^14^. However, owing to the presence of a cutoff energy, harmonic generation in gas can hardly compete with FELs in the high-energy spectral range.

The above-mentioned restrictions can be overcome when applying chirped pulse amplification (CPA)^15^ to FELs that are seeded by an external laser and generate light at the *n*th harmonic of the laser wavelength^16^. The working principle of CPA in seeded FELs^17^,^18^,^19^,^20^ is illustrated in Fig. 1. As in the case of classical lasers, the technique relies on stretching the seed pulse in time by means of a linear frequency chirp before amplification. This virtually allows to extract energy from the whole electron bunch, substantially enhancing the FEL pulse energy at saturation. Moreover, as shown below, the bandwidth of a seeded FEL operated in CPA mode increases with the harmonic number *n*. Such an increase can be ‘sustained' by properly chirping in energy the electron beam, to match the FEL resonant condition. This allows one to circumvent the constraint due to the finite FEL gain bandwidth, removing the limit on the shortest FEL pulse duration reachable with an optical compressor^21^,^22^.

As demonstrated in the Methods, in the presence of a significant frequency dispersion on the seed pulse, the FEL pulse bandwidth, (Δ*ω*)_FEL_, scales according to the relation


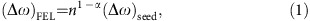


where (Δ*ω*)_seed_ is the seed pulse bandwidth and *α* (positive and smaller than 1/2) is a factor depending on the FEL operating regime. In the following, we will focus on the case of an FEL in moderately saturated regime, corresponding to *α*≃1/3 (ref. 23). Equation (1) points out an important difference between CPA in solid-state lasers and in seeded FELs. Indeed, although in standard lasers the spectral content of the generated light is fundamentally identical to that of the input pulse, in seeded FELs the bandwidth of the output emission can be significantly larger than that of the seed. It is noteworthy that the broadening of the FEL pulse bandwidth relative to that of the seed pulse results from two competing phenomena. On the one hand, the frequency up-conversion process increases the FEL pulse bandwidth by a factor *n* relative to the bandwidth of the seed. On the other hand, this effect is partially counteracted by the dynamic reduction of the FEL pulse duration due to the nonlinear laser–electron interaction within the modulator, represented by the factor *α* (see Methods).

Equation (1) allows us to derive the shortest pulse duration, 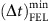, which can be obtained after compressing the FEL pulse:


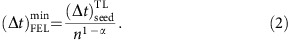


Here 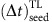 is the seed pulse duration at the transform limit (that is, in the absence of chirp).

In the following, we present the experimental implementation of CPA on a seeded extreme-ultraviolet (XUV) FEL.

## Results

### The setup

**Figure 1:**
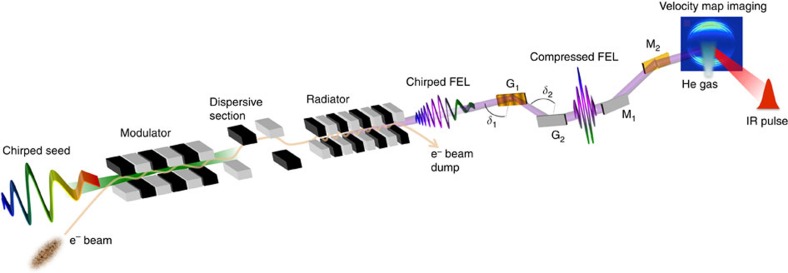
Scheme of a seeded free-electron laser in CPA mode. When operated in CPA regime, the FEL is seeded with a Gaussian laser pulse carrying a linear frequency chirp. The seed interacts with electrons in a short undulator (the modulator). The resulting electron-beam energy modulation is transformed into a density modulation (bunching) when the electrons cross the magnetic field generated by a dispersive section. The bunching has a significant harmonic content at the frequency of the seed, *ω*_seed_, and at its harmonics. Finally, the modulated electrons are injected into a long undulator (the radiator), which is tuned to the *n*th harmonic of the seed. In the radiator, electrons emit coherently at the frequency *ω*_FEL_=*nω*_seed_. Under proper conditions, the frequency chirp of the seed is transmitted to the FEL harmonic pulse generated at the end of the radiator and can then be compensated by an optical compressor. The compressor includes four optical elements: two gratings (G1 and G2) in classical diffraction geometry and two plane mirrors (M1 and M2), which steer the beam back to its original propagation axis. After the exit of the compressor, the FEL beam is directed towards the experimental chamber of the FERMI Low Density Matter beamline (see: http://www.elettra.eu/lightsources/fermi/fermi-beamlines/ldm/ldmhome-page.html) where the FEL pulse duration is measured using a cross-correlation scheme. In the latter, the atoms of a He gas are photo-ionized by the FEL, assisted by a synchronized infrared laser pulse (see Fig. 2). A raw image is shown of a He photo-electron distribution acquired with a VMI spectrometer.

The CPA scheme was tested at the FERMI FEL facility in Trieste^5^. The seed was provided by the third harmonic of a Ti:Sapphire laser (*λ*_seed_=261 nm). The seed-pulse full width at half-maximum (FWHM) bandwidth was 0.7 nm and its initial (before stretching) FWHM duration 170 fs. It is noteworthy that, due to nonlinear effects occurring during harmonic generation and transport to the undulator entrance, the initial seed pulse was not transform limited. Indeed 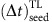
=145 fs. We will further comment on this below. The FEL was tuned to 37.3 nm (*n*=7). The compressor, shown in Fig. 1, consists of two plane gratings with uniform line spacing used at grazing incidence in classical diffraction geometry^24^. The compressor action induced a negative chirp compensating the positive chirp of the FEL pulse. The measured transmission efficiency was ∼5%.

**Figure 2:**
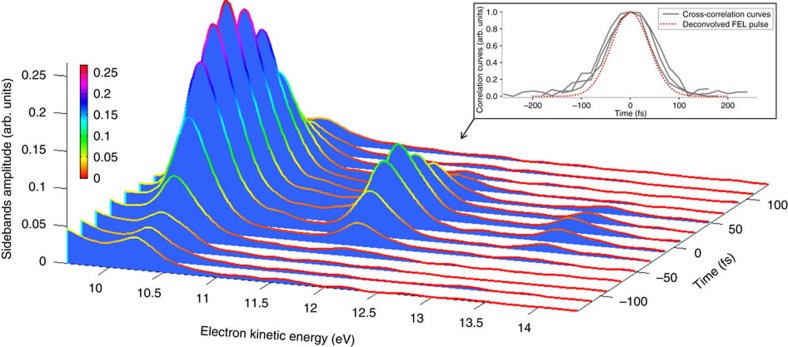
Measurement of the FEL pulse duration. Photo-electron spectra, obtained by inverting the electron distributions acquired with the VMI spectrometer (see Fig. 1), as a function of the delay between the FEL and infrared pulses. The reported measurement refers to the ‘standard' working conditions, that is, no stretching of the seed, no FEL compression. For the sake of visualization, only the first, second and third sidebands (normalized to the main line, associated to the direct photoemission process) are plotted. The inset shows three independent cross-correlation curves obtained by plotting the area under the peak of the second sideband as a function of the FEL-infrared delay. Also shown (dotted curve) is the deconvolved FEL pulse, which has an estimated duration of about 91 fs.

The FEL pulse duration was measured using a cross-correlation scheme^25^, which relies on the ionization of a He gas sample by the FEL in the field of an intense infrared laser with variable time delay and known duration (90 fs, FWHM). A typical image of a He photo-electron distribution, acquired with a velocity map imaging (VMI) spectrometer, is shown in Fig. 1. By inverting the VMI image and integrating over the angular dependence of the electron emission, one gets the photo-electron energy spectrum, which consists of a main line, associated to the direct photoemission process, and of sideband lines, indicating the interaction with the infrared field. The latter are sensitive to the temporal overlap of the FEL and infrared pulses, see Fig. 2. The cross-correlation curves associated to different sidebands, from which one can deconvolve the FEL pulse profile, are obtained by integrating the electron signal over all emission angles and plotting the area under the corresponding peaks as a function of the FEL-infrared delay (see Fig. 2 and Methods).

### The measurements

As a first step, we characterized the FEL spectrum and pulse duration in the standard working conditions, that is, no stretching of the seed and no FEL compression. The measured FWHM spectral width and pulse duration were, respectively, 5.2 × 10^13^ rad s^−1^ (3.8 × 10^−2^ nm) and 91 fs (see inset of Fig. 2), with fluctuations between consecutive measurements of the order of few percent. It is worth noting that the corresponding time-bandwidth product is a factor of 1.7 above the transform limit. Such a deviation is due to an unwanted chirp on the FEL pulse^23^ that results from the combination of two effects: the above-mentioned initial chirp on the seed, which is expected to provide the prevalent contribution and a residual quadratic chirp on the temporal profile of the electron-beam energy. All in all, the measured pulse duration is in very good agreement with the one predicted by the theory^23^ (see Methods), that is ≃89 fs.

**Figure 3:**
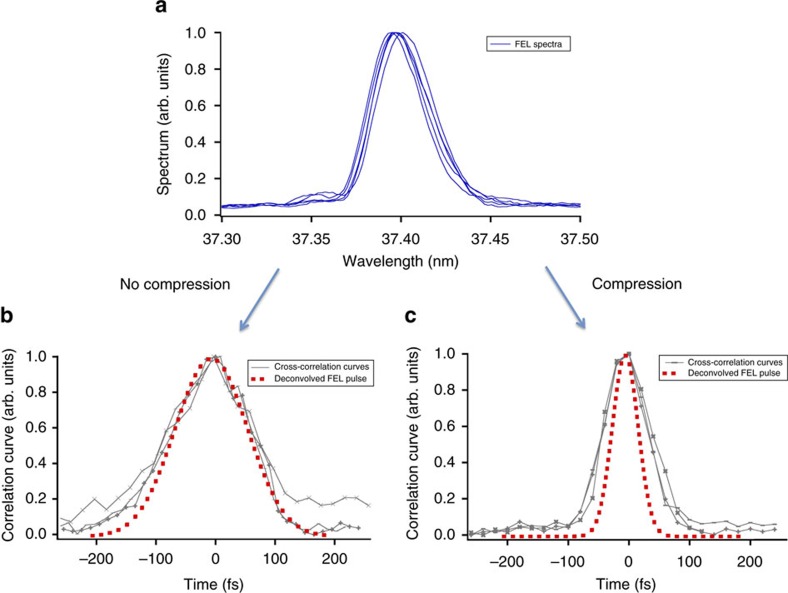
FEL spectro-temporal characterization. (**a**) Normalized single-shot spectra of the free-electron laser (FEL) pulse generated after stretching the seed pulse. (**b**) Three cross-correlation curves associated to the second sideband (see Fig. 2) for three independent FEL-infrared delay scans. The dotted curves represent the deconvolved FEL pulse (assumed to be Gaussian). (**c**) Same as **b** for the FEL operated in CPA mode and the grating angles optimized for maximum compression. As can be seen, the correlation curves from independent scans are quite similar to one another. Moreover, the analysis of the cross-correlation curves associated to the third sideband gives similar results (see Fig. 4). This strongly supports the reliability of the reported results.

**Figure 4:**
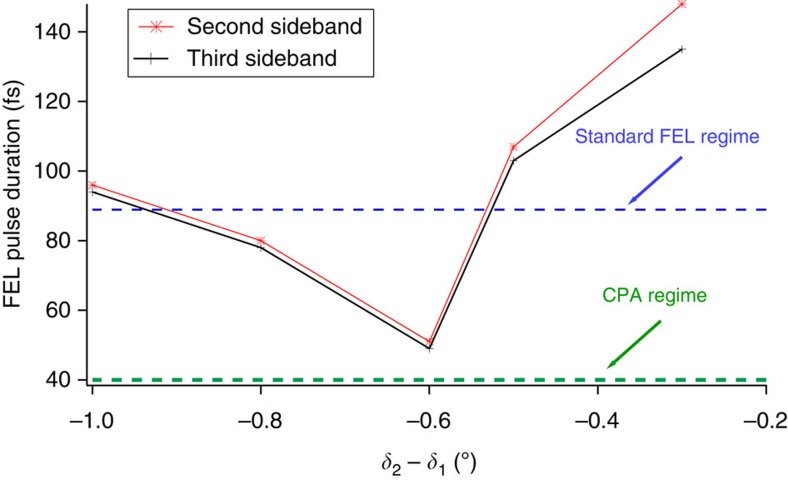
Compression of FEL pulse versus compressor setting. Measured pulse duration as a function of the difference between the diffraction angles of the compressor gratings. Two sets of data are reported, obtained by the analysis of the second and third sideband, respectively, of the photoelectron energy spectrum. The two horizontal dashed lines correspond to the pulse duration expected when the FEL is operated in standard (no seed stretching, no FEL compression) or CPA regime.

Then, to enable the CPA regime, we induced a positive linear frequency chirp in the seed pulse and stretched it up to 290 fs. In these conditions, we characterized the FEL pulse before and after compression. Five independently measured single-shot spectral profiles are shown in Fig. 3a. The average FWHM spectral width is 6.05 × 10^13^ rad s^−1^ (4.46 × 10^−2^ nm), with fluctuations between consecutive measurements of the order of few per cent. This result is close to the theoretical value predicted by equation (1), that is, 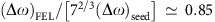. Three independently measured cross-correlation curves, associated to the second sideband of the photo-electron spectrum, are shown in Fig. 3b for the non-compressed case and in Fig. 3c for the case of maximum compression. Also shown (dotted curves) are the deconvolved FEL pulses. The obtained FWHM pulse duration for the case of no-compression is ∼143 fs (with fluctuations of few per cent), which is again in satisfactory agreement with the theoretical expectation of ∼152 fs. The increase of the FEL pulse bandwidth with respect to that of the seed, which is the essence of CPA in seeded FELs, allowed us to obtain, after compression, a significant shortening of the FEL pulse with respect to the no-CPA case. For the reported case, see Fig. 3c, 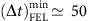 fs. This value is quite close to the one predicted by equation (2), that is ∼40 fs. The measured time-bandwidth product is now only a factor of 1.1 above transform limit. This remarkable result shows that the CPA technique is able to compensate not only the linear frequency chirp in the FEL pulse induced through the seed control, but also the unwanted residual generated by other sources, such as the seed transport and the quadratic curvature of the electron-beam energy profile. The effect of the compressor setting is shown in Fig. 4, in which the measured pulse duration is reported, as a function of the difference between the FEL incident angles on the two gratings (*δ*_1_ and *δ*_2_ in Fig. 1).

## Discussion

We demonstrated the possibility of carrying out CPA in an extreme-ultraviolet seeded FEL. The technique, which can be extended to FELs based on self-amplified spontaneous emission^26^,^27^ and to plasma amplifiers^28^, allowed us to achieve a relevant reduction of the FEL pulse duration, with respect to that obtained in standard operation mode. The present low transmission of the compressor (∼5%) and the relatively small harmonic number (*n*=7) limited the obtained peak power to a fraction of a gigawatt. However, by adopting an off-plane mount geometry of the gratings^24^ (which has a proven peak efficiency up to 70% measured on a single grating^29^), and by choosing *n*>60 (which is feasible using, for example, the FEL-2 configuration at FERMI^30^), the potential is there to produce, with existing technology, coherent few-femtosecond gigawatt laser pulses. In a perspective view, a natural extension of our results may allow to generate fully coherent sub-femtosecond FEL pulses at wavelengths close to the K-absorption edge of oxygen (2.3 nm), paving the way to X-ray imaging experiments with unprecedented temporal resolution in the water window. Based on the reported results, we believe that CPA will soon become an essential tool for existing and future X-ray FELs.

## Methods

### FEL and seed settings

For the reported experiment, the electron-beam energy was tuned to 1.2 GeV, the peak electron current was set to about 500 A and the bunch duration was ∼1 ps. The electron beam profile was close to constant in the region seeded by the laser pulse (that is, few hundreds of femtosecond around the beam centre), with a residual quadratic energy curvature of the order of few MeV ps^−2^.

The seed was provided by the third harmonic of a Ti:Sapphire laser (261 nm, Gaussian spectral profile). In standard operating conditions (no CPA), the seed had a FWHM duration of 170 fs and a FWHM bandwidth of ∼0.7 nm. For the CPA operation, a positive linear frequency chirp was introduced by propagating the third-harmonic pulse through a calcium fluoride plate that stretched the pulse duration to 290 fs. The peak power at the entrance of the modulator was ∼250 MW, for both the standard and CPA configurations.

The FERMI FEL modulator has a period of 100 mm and a length of 3 m. The radiator is composed of six 2.4 m-long APPLE-II undulator sections with a period of 55 mm. During the reported experiment, the strength of the dispersive section was about 50 μm.

### The optical compressor

The compressor consists of four optical elements: two gratings (G1 and G2 in Fig. 1) and two plane folding mirrors (M1 and M2 in Fig. 1), which steer the beam back to its original propagation axis. The control of the optical delay between different FEL spectral components is achieved through the variation of the FEL incident angles (*δ*_1_ and *δ*_2_ in Fig. 1) on the gratings. The latter can be varied independently. This allows the compensation of the wavefront tilt due to the divergence of the FEL beam. The gratings are placed at a distance of 400 mm, have a groove density of 600 gr mm^−1^ and a blaze angle of 2°. The compressor is hosted in a ultra high-vacuum chamber. The optics are moved by external stepper motors. The angular resolution is 65.8 μrad per step in a full step mode; controllers are capable of a 1/8-step resolution. The angles *δ*_1_ and *δ*_2_ may be selected in the range between −0.5° and 14.5°.

### Measurement of the FEL pulse duration

The FEL pulse duration was measured using a cross-correlation scheme, which relied on the ionization of a He gas sample by the FEL in the field of an intense infrared laser pulse with variable delay and known duration (90 fs, FWHM). The photo-emitted electrons were acquired using a VMI spectrometer. To reconstruct the electron energy spectrum, the VMI images have been inverted using the programme MEVIR (maximum entropy velocity image reconstruction^31^). The cross-correlation curves associated to different sidebands are obtained by integrating the electron signal over all emission angles and plotting the area under the corresponding peaks (see Fig. 2), as a function of the FEL-infrared delay. Neglecting the time jitter between the FEL and the infrared laser, assuming Gaussian temporal profiles and moderate infrared energies (no saturation of sidebands intensities), the FEL pulse duration can be obtained from the following relation: 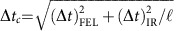, where Δ*t*_*c*_ is the FWHM of the cross-correlation curve, (Δ*t*)_IR_ is the width of the infrared probe pulse and 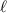 is the sideband order.

### Theoretical framework of CPA in seeded FELs

Consider a Gaussian chirped laser pulse, whose electric field in the frequency domain reads 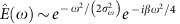. Here *σ*_*ω*_ is the (r.m.s.) laser bandwidth and *β* is the so-called group delay dispersion^32^, which is used in the following to quantify the frequency dispersion. The Fourier transform of 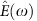 is 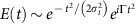. The coefficient of the quadratic temporal phase, Γ, and the (r.m.s.) laser pulse duration, *σ*_*t*_, are related to *σ*_*ω*_ and *β* through the following relations: 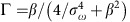 and 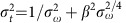. Based on the previous relations, two extreme regimes can be distinguished: (a) for 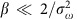: 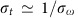 and (b) for 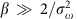: 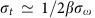. The latter regime is the one suitable for CPA. It is worth noting that the previous relation is valid also for non-Gaussian pulse profiles, due to the equivalence between the spectral and temporal profiles (both in amplitude and phase) in the case of strongly chirped pulses^33^.

Consider now the case of a seeded FEL. For a Gaussian seed laser pulse, the FEL is expected to have a quasi-Gaussian pulse profile. According to the literature^23^, the duration of the FEL pulse in standard operating conditions (no CPA) is 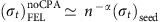, where (*σ*_*t*_)_seed_ is the seed pulse duration and *α* (positive and smaller than 1/2) is a factor depending on the regime in which the FEL is operated. As a consequence of the frequency up-conversion, the temporal phase profile of the FEL pulse is *n* times that of the seed. On the basis of the above, one can easily show that, if the seed is characterized by a strong chirp (and the longitudinal electron-beam energy profile can be assumed to be almost flat), the same holds for the FEL^33^. In this case, always in the high-chirp regime, the amount of group delay dispersion to be compensated by the optical compressor, *β*_FEL_, is related to *β*_seed_ by the following relation: 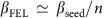. By combining the previous equations, the fundamental relations (1) and (2) reported in the main text can be obtained. It is noteworthy that, for the sake of comparison with experimental results, the latter are expressed in FWHM for the spectral (Δ*ω*) and temporal (Δ*t*) powers.

### Data availability

All relevant data contained in this manuscript are available from the authors.

## Additional information

**How to cite this article:** Gauthier, D. *et al*. Chirped pulse amplification in an extreme-ultraviolet free-electron laser. *Nat. Commun.* **7,** 13688 doi: 10.1038/ncomms13688 (2016).

**Publisher's note**: Springer Nature remains neutral with regard to jurisdictional claims in published maps and institutional affiliations.
